# Providing office workers with height-adjustable workstation to reduce and interrupt workplace sitting time: protocol for the Stand Up for Healthy Aging (SUFHA) cluster randomized controlled trial

**DOI:** 10.1186/s13063-023-07407-9

**Published:** 2023-06-06

**Authors:** Pedro B. Júdice, Hélio Silva, Sabrina C. Teno, Patrícia Monteiro, Marlene N. Silva, Eliana V. Carraça, Inês Santos, Sara Pereira, Filipe Luz, Patrícia C. Viegas, Jorge Oliveira, Isabel F. Santos, António L. Palmeira

**Affiliations:** 1 CIDEFES, Universidade Lusófona, Lisbon, Portugal; 2 Escola de Psicologia e Ciências da Vida, Universidade Lusófona, Lisbon, Portugal; 3 Programa Nacional para a Promoção da Atividade Física- Direcção-Geral da Saúde, Lisbon, Portugal; 4 Laboratório de Nutrição, Faculdade de Medicina, Centro Académico de Medicina de Lisboa, Universidade de Lisboa, Lisbon, Portugal; 5 Instituto de Saúde Ambiental (ISAMB), Faculdade de Medicina, Universidade de Lisboa, Lisbon, Portugal; 6 CIFI2D, Faculty of Sport, University of Porto, Porto, Portugal; 7 Hei-Lab - Universidade Lusófona, Campo Grande, Lisbon, Portugal; 8 Center for Other Worlds - Universidade Lusófona, Campo Grande, Lisbon, Portugal

**Keywords:** Sitting time, Sedentary behavior, Sit-stand desk, Motivational nudges, Contextual modification, ActivPAL, Standing time

## Abstract

**Background:**

Sedentary behavior (SB) has been linked to several negative health outcomes. Therefore, reducing SB or breaking up prolonged periods of SB improves functional fitness, food consumption, job satisfaction, and productivity. Reducing SB can be achieved by introducing a health-enhancing contextual modification promoted by a sit-stand desk in the workplace. The primary goal will be to test the effectiveness of this intervention in reducing and breaking up SB, while improving health outcomes in office-based workers during a 6-month intervention.

**Methods:**

A two-arm (1:1), superiority parallel-group cluster RCT will be conducted to evaluate the effectiveness of this intervention in a sample of office-based workers from a university in Portugal. The intervention will consist of a psychoeducation session, motivational prompts, and contextual modification promoted by a sit-stand desk in the workplace for 6 months. The control group will work as usual in their workplace, with no contextual change or prompts during the 6-month intervention. Three assessment points will be conducted in both groups, pre-intervention (baseline), post-intervention, and a 3-month follow-up. The primary outcomes include sedentary and physical activity-related variables, which will be objectively assessed with 24 h monitoring using the ActivPAL for 7 days. The secondary outcomes include (a) biometric indices as body composition, body mass index, waist circumference, and postural inequalities; and (b) psychosocial variables such as overall and work-related fatigue, overall discomfort, life/work satisfaction, quality of life, and eating behavior. Both the primary and secondary outcomes will be assessed at each assessment point.

**Discussion:**

This study will lean on the use of a sit-stand workstation for 6 months, prompted by an initial psychoeducational session and ongoing motivational prompts. We will aim to contribute to this topic by providing robust data on alternating sitting and standing postures in the workplace.

**Trial registration:**

The trial was prospectively registered, and the details are at: https://doi.org/10.17605/OSF.IO/JHGPW; Registered 15 November 2022. OSF Preregistration.

## Administrative information

Note: the numbers in curly brackets in this protocol refer to SPIRIT checklist item numbers. The order of the items has been modified to group similar items (see http://www.equator-network.org/reporting-guidelines/spirit-2013-statement-defining-standard-protocol-items-for-clinical-trials/).

**Table:** 

Title {1}	Providing office-workers with height-adjustable workstation to reduce and interrupt workplace sitting time: Protocol for the Stand Up for Healthy Aging (SUFHA) cluster randomized controlled trial
Trial registration {2a} and {2b}.	Trial registration: https://doi.org/10.17605/OSF.IO/JHGPW; Registered 15 November 2022. OSF Preregistration.
Protocol version {3}	11 February 2023, version 1.
Funding {4}	This study was funded by the ILIND “Fazer+” scientific program (FAZER+/ILIND/CIDEFES/1/2022). The funder has no role in the study in terms of the design, data collection, management, analysis, and interpretation.
Author details {5a}	1 CIDEFES, Universidade Lusófona, Lisboa, Portugal 2 Escola de Psicologia e Ciências da Vida, Universidade Lusófona, Lisboa, Portugal 3 Programa Nacional para a Promoção da Atividade Física- Direcção-Geral da Saúde, Lisboa, Portugal 4 Laboratório de Nutrição, Faculdade de Medicina, Centro Académico de Medicina de Lisboa, Universidade de Lisboa, Lisboa, Portugal 5 Instituto de Saúde Ambiental (ISAMB), Faculdade de Medicina, Universidade de Lisboa, Lisboa, Portugal 6 CIFI2D, Faculty of Sport, University of Porto, Porto, Portugal 7 Hei-Lab - Universidade Lusófona, Campo Grande, Lisboa, Portugal 8 Center for Other Worlds - Universidade Lusófona, Campo Grande, Lisboa, Portugal
Name and contact information for the trial sponsor {5b}	ILIND - Instituto Lusófono de Investigação e Desenvolvimento Contact: ilind@ulusofona.pt & 217 515 500 ext 756
Role of sponsor {5c}	The funder has no role in the study in terms of the design; collection, management, analysis, and interpretation of data; writing of the report; and the decision to submit the report for publication.

## Introduction

### Background and rationale {6a}

There is a strong interest in capitalizing on years of health and reducing the period of disability in adults to enable well-being in aging. In line with this, it is necessary to further assess the impact of reducing sedentary behavior (SB) or breaking up prolonged bouts of SB on relevant health outcomes, quality of life, and productivity throughout the lifespan [1].

The evidence supporting the importance of reducing SB is plentiful; high levels of SB are linked with physical inactivity (i.e., not attaining the recommended amount of physical activity (PA), which in turn has been associated with obesity [2], metabolic disorders [3, 4], and all-cause mortality [5, 6]. In addition to physical inactivity, the negative role of excessive SB on health has been identified, with SB being linked to all-cause and cardiovascular diseases (CVD) mortality [7, 8] and to decreased life expectancy, with 3 h of sitting per day leading to a life expectancy decrease of 2 years [9, 10]. Martinez-Gomez and colleagues showed that older adults who spent less than 8 h sitting/day had a lower risk of all-cause mortality, when compared with their sedentary peers [11]. A prospective study found that during 6.8 years, greater sitting time (≥12 vs <5 h/day) was associated with increased risk for all-cause and CVD mortality [7], and that in less-active adults, replacing 1 h/day of sitting with an equal amount of activity was associated with lower all-cause mortality for both exercise and non-exercise activities. Recently, it was found that replacing SB with light-intensity physical activity (LIPA) may protect against cognitive decline by reducing glycemic variability [8] and increasing cerebral blood flow [12].

In addition, SB and PA seem to be independently related to functional fitness in older adults [13]. Interestingly, breaking up SB seems to be even more important than total SB; it has been associated with lower abdominal obesity in older women [14] and each transition between sitting and standing (i.e., break in sedentary time) entails high energy expenditure [15], suggesting that prolonged SB must be avoided [16]. Breaking up SB is also positively associated with physical function [17] and the capacity to perform activities of daily living in older adults [18]. Moreover, in patients with type two diabetes, breaking SB more often was associated with higher levels of brain-derived neurotrophic factor (BDNF), a cognitive and memory indicator, which is additionally responsible for fat oxidation in the muscle [19].

SB has also been linked to food consumption across the lifespan, particularly to spontaneous compensations in habitual nutrition (with greater consumption of energy-dense snacks and sugar sweetened beverages and fewer fruits and vegetables) [20]. Although evidence is still in its early stages, a recent study by Grant and colleagues indicates that displacing SB with LIPA improves dietary quality in older females and that SB fragmentation appears advantageous for various dietary outcomes [21]. These findings suggest that replacing SB with a variety of activities is important, regardless of their intensity; however, the next step is to confirm these hypotheses in experimental studies.

The World Health Organization (WHO) data indicates that workers represent approximately half the global population and that most of the population spends an average of one-third of their adult life at work—in jobs that augment SB by sitting and commuting. As such, employers have an opportunity to create supportive environments to encourage workers to reduce SB. Consequently, researchers should prioritize the study of the effectiveness of reducing SB’ interventions at the organizational level [22, 23]. Indeed, office-based workers are highly sedentary due to the job demands (e.g., meetings, working with a computer), making them a key target group for intervention [24].

In office-based workers, higher SB has been associated with lower job satisfaction and greater fatigue, when compared with lower SB. Results offer promising support that less sitting time is associated with positive outcomes that do not seem to come at the expense of productivity [25]. Thus, the office is a key setting to reducing prolonged SB [26, 27], and this is an important consideration in the context of the duty of care obligations of employers to ensure, so far as it is reasonably feasible.

Several sit-stand desk-based RCT interventions have been performed worldwide. In the last decade, most of them took place in Oceania, with 4 being held in Australia [28–31] and 3 in New Zealand [32–34]. Five interventions were held in the United Kingdom (UK) [35–39] and 2 interventions in Canada [40, 41]. The United States of America (USA) contributed with 5 sit-stand desk-based RCTs [12, 42–45] and Japan with 1 intervention [46]. Notice that 14 out of the 23 RCTs were performed in countries belonging to the Commonwealth, and 19 out of 23 in English-speaking countries. In Europe, there is a lack of interventions using sit-stand desks, with only 1 in Finland [47], 1 in Austria [48], and 1 in Switzerland [49]. There is a need for more evidence on the implementation acceptance and effectiveness of these new approaches in the work environment, especially in non-English-speaking countries. While several interventions seemed effective at reducing SB in the workplace [31, 50–58], there is no information from Portuguese-speaking countries, when considering the adult population, which deserves our attention.

### Objectives {7}

This paper describes the protocol for a cluster randomized controlled trial (RCT) the Standing Up for Healthy Aging (SUFHA), which aims to evaluate the change in sedentary patterns (i.e., quantity and accumulation pattern) resulting from the introduction of a health-enhancing contextual modification (i.e., sit-stand desk) in the workplace, compared to a control group after 6 months of intervention (i.e., effectiveness). Secondary outcomes are body composition, eating patterns, health and work-related outcomes, skeletal muscle discomfort, well-being, and quality of life. Additionally, we aim to crossover the intervention and test the impact of 3 months without the sit-stand desks on sedentary patterns in the intervention group and the impact of 3 months of sit-stand desks in the control group, and the level of acceptance regarding the use of these sit-stand desks in both groups.

### Trial design {8}

A two-arm (1:1), superiority parallel-group cluster crossover RCT will be undertaken to evaluate the SUFHA intervention. The Consolidation Standards of Reporting Trials (CONSORT) statement for cluster RCTs will be used to conduct, analyze, and report this study.

### Patient public involvement {8a}

The Principle Investigator (PI) will regularly meet with the researchers and students involved in the study on a weekly basis and with the PIs of the projects under CIDEFES every 2 weeks. During these meetings, senior researchers will discuss the main issues of SUFHA and how experiences and proposals from participants should be taken into consideration during the decision-making process for final intervention refinement. As this is a low-contact intervention, we did not consider other participatory research design methods.

## Methods: participants, interventions, and outcomes

### Study setting {9}

Office-based workers will be recruited via university advertisements (e.g., posters, banners, invitation emails). The ones interested and fulfilling the inclusion criteria will be contacted to participate in the trial with sit-stand desks at work and their experiences on barriers and facilitators of use collected (via focus group) to delve deeper into their motivations or doubts related to the study. A website was created so that the participants can have more detail on the RCT itself, evaluations, expectations, and a tool for disseminating strategies to prompt participants to use the sit-stand desk throughout the intervention (https://sufha.ulusofona.pt).

### Eligibility criteria {10}

The inclusion criteria for this study comprise working at least 0.6 full-time; being more than 20 years old; and spending at least 70% of a working week performing desk-related activities. Participants will not be able to participate if they already use a sit-stand desk, if they do not have a personal space where a sit-stand desk can be assembled, or if they have musculoskeletal disorders/health conditions, making it impossible to work in a standing posture.

### Who will take informed consent? {26a}

The PI of the study will contact all potential participants for a detailed explanation of the trial, as well as provide the link to the website of the study, so that all doubts can be solved prior to the baseline evaluation moment. On the previous day to the baseline measurement, each potential participant will receive an email with informed consent, allowing access to all the corresponding information in advance. On the next day (baseline assessment), one trained evaluator (i.e., Ph.D. candidate with a Master in Exercise and Health and a certified Exercise Physiologist by the Portuguese Government) will answer any questions prior to the potential participants signing the written informed consent.

### Additional consent provisions for collection and use of participant data and biological specimens {26b}

On the consent form, participants will be asked if they agree to use of their data should they choose to withdraw from the trial. Participants will also be asked for permission for the research team to share relevant data with people from the University taking part in the research or from regulatory authorities, where relevant. This trial does not involve collecting biological specimens for storage.

## Interventions

### Explanation for the choice of comparators {6b}

SUFHA is designed for a sample of adult University office workers and will consist of two groups. A waiting-list control group will only attend a psychoeducational session regarding the independent benefits of reducing and interrupting sitting time with standing, with no contextual change during the 6-month intervention (i.e., no access to the sit/stand desk). The intervention group will attend the psychoeducational session and will be provided with the opportunity to have a sit-stand desk in their workplace, allowing them to work seated or standing and change between these postures as many times as they desire for 6 months.

After the intervention period, the waiting-list control group will have access to the sit-stand desk.

### Intervention description {11a}

SUFHA will be delivered over 6 months, with another 3 months of follow-up. Given the relative lack of literacy concerning the independent effects of SB in health (i.e., most health campaigns have been focused on raising PA levels) [59], both the intervention and control groups will attend an initial psychoeducational session regarding the independent benefits of reducing and interrupting sitting time with standing and other activities. Besides raising information and literacy, this type of strategy (i.e., educational seminar) has been found to facilitate behavior change in a similar setting [50]. The intervention group will be provided the opportunity to work on a sit-stand desk (Vinsetto, Model 923-043) for 6 months, while the control group will remain on a traditional sitting desk. The sit-stand desks allow the employees to choose if they want to work while sitting or standing and change between postures as often as they desire and have been found to be crucial for the aimed behavioral change [50].

As mentioned above, the intervention will comprise an initial session given by a specialist in this area of research (i.e., Assistant Professor in a Sports Science Faculty with a Master in Exercise and Health, a Ph.D. and a Post-doctoral training within the sedentary behavior research area, with over 50 publications in this field and with many years of experience with observational and experimental studies within the Scopus of sedentary behavior) concerning the benefits of reducing sitting time and interrupting it with standing activities, providing information and practical tips on how to reduce sedentary time and use the sit-stand desk. Before the beginning of the intervention and considering the randomization process, a visit will be made to the work site of the participants allocated to the intervention group, to assemble the sit-stand desks, without any associated costs. Desk mounts involve a device that is installed on top of a conventional workplace desk often by means of a clamping arm. The device facilitates regular transitions between sitting and standing postures, predominantly while performing computer-based activities. It can be placed in standing mode via an easy upward pulling motion that lifts the display unit(s) and objects placed on the work surfaces. To enhance desk use, participants will also be given the following: (i) a demonstration of how their sit-stand desk works; (ii) tailored information on the correct ergonomic posture; (iii) and individualized guidance on gradually building up standing time. This information will then be written down on a flyer allowing the participants to read it anytime they feel necessary. Participants will afterward be able to use their sit-stand desk for the following 6 months.

There is evidence that temporary context changes, such as holidays, threaten to derail newfound routines, such as standing more often in the workplace [39]. Furthermore, after the intervention period, participants will no longer have the sit-stand desk. Thus, to enhance motivation and self-regulation for internalizing the new routines of breaking up sitting time, intervention participants will also be provided motivational nudges throughout the intervention. Delivery of these nudges will vary from 4 times in the first month, to 2 times in the second and third months, and monthly until the end of the intervention. These motivational nudges will be delivered in different formats, designed to be appealing and engaging (e.g., animated short videos containing simple tips on different forms of breaking sitting time and benefits of that); social support and social modeling will also be enhanced via these nudges (e.g., including testimonials from study participants willing to share their ongoing experience, including photos and short videos using the desk) allowing to support relatedness, perceived competence, and autonomy to use the desk. These are the three basic psychological needs supporting autonomous motivation according to Self-Determination Theory [60]. This type of motivation, in turn, has been shown to allow the integration of new health behaviors in the long term while also enhancing well-being [61].

To determine if the outcomes’ changes observed during the 6 months of work can be maintained in a context without the stimulus (i.e., no sit-stand desk), the intervention group will be followed for 3 months, in which they will be without the sit-stand desks. By the end of the intervention period and at the end of the follow-up, assessments will be conducted, and feedback provided to all participants assuring valuable information regarding participants sedentary patterns, body composition, eating patterns, health and work-related outcomes, fatigue, presenteeism, job satisfaction, well-being, and quality of life, and information on how these have changed over the course of the study.

### Criteria for discontinuing or modifying allocated interventions {11b}

By the end of the randomization process that will allocate all participants to one of the two groups (i.e., control vs intervention), modifying allocated interventions for a given trial participant will not be allowed, except if the participant request is based on a specific health condition that interferes with the study. But these situations will be kept to a minimum.

### Strategies to improve adherence to interventions {11c}

Initially, a psychoeducational session regarding the independent benefits of reducing and interrupting sitting with standing will be given. Besides raising information and literacy, this type of strategy (i.e., educational seminar) has been found to facilitate behavior change in a similar setting [50]. Furthermore, this psychoeducational session will also be given to the control group, highlighting the importance of their participation in the trial (description of how important RCTs are for science will be embedded in the information provided) and reducing the risk of dropout. Control group will also be provided with the intervention (waiting-list group) after the end of the intervention group; thus, it is important to raise early awareness (via the initial session) of the importance of these types of interventions. To enhance desk use, participants will also be given the following: (i) a demonstration of how their desk works; (ii) information on the correct ergonomic posture; and (iii) guidance on gradually building up standing time, as evidence suggests that behavioral feedback and regular contact with research staff are key elements of change [50].

Finally, a theory-based system of prompts will be developed and offered to the participants, assuring contact and motivational nudges throughout the intervention period (for detail, see “Intervention description {11a}”).

In order to gain understanding of participant experiences with the intervention program, two courses of action are planned, both allowing not only to gain in-depth understanding of participants’ barriers and facilitators, but also to help in the refinement of the intervention for future deliveries:

(i) Focus groups with a subsample of participants, at the middle of the intervention period, will also be conducted, allowing the participants the opportunity to voice their experiences concerning the use of the new desk (e.g., interest, utility vs. the barriers for use), and interest and usefulness of the intervention contents on the website and team support (e.g., sufficient? Other contents/needs to be addressed)
(ii) Insights from the participants will be collected after the initial psychoeducation session, via an open-ended form. This will inform future applications of the intervention. The open-ended form will allow us to check if the information was useful and stimulating and if other contents should be addressed.

### Relevant concomitant care permitted or prohibited during the trial {11d}

Apart from the type of desks at the workplace, no control will exist regarding potential extra strategies that both groups can develop outside this context. However, the participants will be asked about other potential strategies they developed during the study on the second evaluation moment.

### Provisions for post-trial care {30}

We do not expect any harm from this intervention, as we are providing the opportunity for both groups to experience working on a sit-stand desk, without a mandatory amount of change. Regulation of the amount of sitting time replaced by standing will be settled by each participant, informed by information provided in terms of safety and progression given in the initial psychoeducational session and at the time of desk installation. To enhance the internalization of the new habits of breaking sitting time potentially raised by the sit-stand desk, motivational prompts to enhance autonomous motivation and self-regulation in the long term (after intervention-end) will also be provided.

### Outcomes {12}

#### Primary

The primary outcomes of SUFHA are the sedentary and PA-related outcomes (e.g., minutes of SB/day, minutes of sitting time/day, minutes of standing time/day, sit-to-upright transitions/day, number of steps, among others), which will be objectively assessed with 24-h monitoring using the ActivPAL for 7 consecutive days. Changes in these parameters are the main goal of the current RCT (i.e., effectiveness). More details regarding the assessment that generate these outcomes can be found in the “Data collection and management” section. These outcomes will be measured at baseline (i.e., a week within the 30 days prior to the installation of the sit-stand desks), 2nd moment (i.e., final month of intervention), and 3rd moment (i.e., final month of follow-up). Additionally, in the third month of intervention, all participants will be challenged to use the ActivPAL. Although this assessment moment is not mandatory, we expect a significant adherence to this challenge.

#### Secondary

The secondary outcomes of SUFHA are body composition (i.e., % of fat mass and fat-free mass, both estimated based on bioimpedance), body mass index (BMI), and waist circumference, all objectively assessed. Postural inequalities based on the analysis of two photos. The Data collection and management section provide more details regarding the assessments that generate these outcomes.

SUFHA also aims to explore secondary outcomes, such as eventual changes in medication, and some psychosocial outcomes, such as overall and work-related fatigue, overall discomfort, life/work satisfaction that comprises four domains (i.e., physical, psychological, environmental, and social), and quality of life. Eating-related habits/behaviors will also be assessed as secondary outcomes, generating individual parameters and an overall score of adherences to the Mediterranean diet and specific behaviors such as breakfast consumption, skipping meals, fast food consumption, homemade meals, take-away meals, and finally eating behavior traits (i.e., emotional eating, intuitive eating, eating restriction, flexible restraint, external eating, and uncontrolled/disinhibited eating). All secondary outcomes will also be measured at baseline (i.e., month before starting the intervention), 2nd moment (i.e., final month of intervention), and 3rd moment (i.e., final month of follow-up).

### Participant timeline {13}

**Fig. 1:**
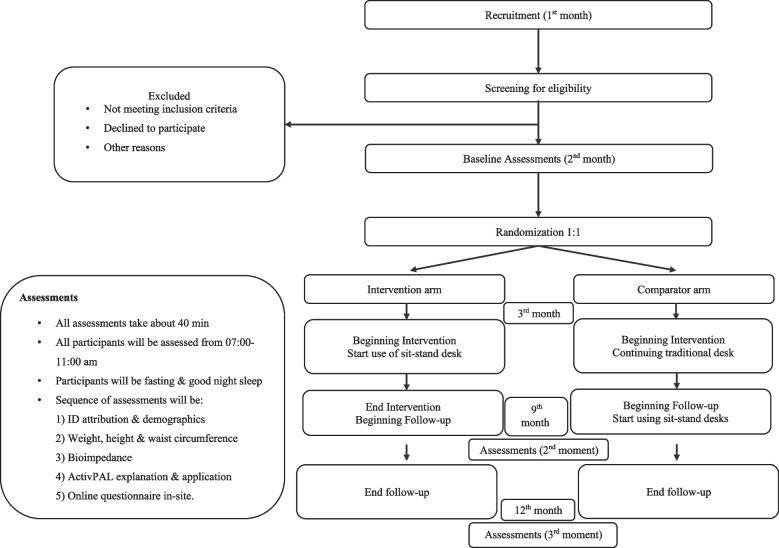
Flow diagram of enrolment, interventions, assessments, and visits for participants

The time schedule of enrolment, interventions, assessments, and visits for participants are presented in a diagram (see Fig. 1). All participants will enter the study and be assessed at the same time points.

### Sample size {14}

For cluster size and number calculation, we assumed that participants within the same workplace (cluster) would be independent, and therefore, an intra-cluster correlation of 0.01 will be used. Considering the main outcome of the present intervention (i.e., sitting time) and using an effect size of 0.80, the total number of participants for between-group analyses (control and intervention groups) with a power of 0.80 and a significance level of 0.05 (two-tailed test) would be 34 participants. Assuming a 20% dropout, we will conservatively recruit a total sample size of 40 participants (i.e., 20 controls and 20 in the intervention group).

### Recruitment {15}

We will use different recruitment strategies to match preferences. These will include institutional emails and a project website created with all the information about the project. Also, posters will be created by a professional design team for recruitment and spread out on the University campus. Finally, face-to-face may complement recruitment to attain the aimed number of participants. Based on the average interest rates reported in previous studies assessing the impact of sit-stand desks on SB, the expected proportion of participants interested in taking part in a trial of sit-stand desks at work is 37% of the office workers [62]. All the participants indicating an interest in taking part in the trial will be visited by a researcher who will assess their workspaces for desk installation suitability and 40 participants will be randomized to either the use of sit-stand desks at work for 6 months (i.e., intervention group) or a control group that will only receive the sit-stand desks after the intervention period (i.e., during the 3 months of follow-up).

## Assignment of interventions: allocation

### Sequence generation {16a}

After baseline data collection, cluster randomization using a list randomizer (www.random.org) will be performed by a researcher not involved in recruitment or data collection. The randomization and group allocation process will be made using the different sites as the unit of randomization, to reduce the risk of the intervention being contaminated. A total of 40 office-based workers from different sites will be randomized into two groups: 20 participants to the control group without sit-stand desks and 20 participants to the intervention group with sit-stand desks at work for 6 months. The randomization will consider that control and intervention groups can be matched in some key characteristics, such as the number of transitions from sit to stand and the overall time spent in SB, the BMI, and age. Finally, the randomization will consider if the participants can observe each other from their workstations. In that case, they will constitute a cluster and be randomized as such instead of being randomized individually.

### Concealment mechanism {16b}

Participants will be randomized using random number generator. The allocation concealment mechanism will be used once the participants will not be informed of the allocation group prior to the intervention, which takes place after all baseline measurements have been completed.

### Implementation {16c}

An independent researcher will generate the allocation sequence. The research team will then enroll participants and assign them to interventions. The participants will be informed about the group they were assigned (i.e., based on a random number generator performed autonomously by an independent researcher) by email.

## Assignment of interventions: blinding

### Who will be blinded {17a}

Baseline assessment will take place prior to randomization; thus, at this point, participants and field work staff will be unaware of group allocation. After baseline, due to the nature of the intervention, it will not be possible to blind participants or staff.

### Procedure for unblinding if needed {17b}

After randomization is performed and when communicating to the participants their group allocation, unblinding will be required. At this time, the trial manager, data coordinator, implementation support facilitators, care home managers, and participants will have access to group allocations.

## Data collection and management

### Plans for assessment and collection of outcomes {18a}

#### Primary data collection

##### Sedentary and PA pattern assessment

We will collect sedentary and PA pattern data derived from an ActivPAL inclinometer/accelerometer monitor (model ActivPAL4; PAL Technologies Ltd., Glasgow, UK), while using the CREA (v1.3) classification algorithm that allows to distinguish non-wear, lying/sitting, standing, cycling, seated during transport, and stepping time as well as the number of steps and stepping cadence. The ActivPAL combines the information from an inbuilt inclinometer and accelerometer and is the reference objective method for monitoring sedentary activity and transitions from and to this behavior among researchers. Due to its position on the thigh, the ActivPAL permits the distinction between standing (vertical position) from sitting or lying (horizontal position), and the accelerometer data allows to distinguish stationary upright activities (i.e., standing) from upright activities with movement (i.e., stepping). The following variables will be calculated from the device:

1. Mean minutes spent sitting,
2. Mean minutes spent standing
3. Mean minutes spent stepping
4. Mean number of sit-to-upright transitions
5. Mean minutes spent in prolonged (e.g., bouts of ≥30 min) sitting
6. Mean minutes spent sleeping

These variables will be calculated across the total waking day. Participants will wear the device on their right thigh 24 h/day for 7 days at baseline, repeating this assessment at the end of the intervention (after 6 months), and after the follow-up period (after 9 months). The 24 h/day wear period will allow to also collect data on sleep quantity.

More information regarding the procedures related to ActivPAL application are stated in the “Procedures” section.

#### Secondary data collection

##### Demographic data

Information on age, sex, country of birth, education, financial status, occupation, and tasks assignment/duties, working hours and years of work, current smoking status, alcohol consumption, presence of chronic disease, usual way of commuting, and structured exercise and SB on week and weekend days will be self-reported trough an online questionnaire. Total use of medication and eventual changes in medications will be collected from the patient records.

##### Body composition and postural inequality assessment

Participants will be weighed to the nearest 0.01 kg while wearing minimal clothes and without shoes, on an electronic scale (TANITA BC-601 scale). Height will be measured to the nearest 0.1 cm with a stadiometer (Seca, Hamburg, Germany). BMI will be calculated as body mass (kg)/height^2^ (m). BMI will be further categorized into normal weight (<25 kg/m^2^), overweight (25–29.9 kg/m^2^), and obesity (>30 kg/m^2^). Waist circumference will be measured with the participants in a standing position, over the naked skin, to the nearest 0.1 cm. The tape is applied horizontally just above the uppermost lateral border of the right ilium at the end of a normal expiration. The mean of two measurements will be considered. If the two measurements differ by more than 1 cm, a third measurement will be performed, and the two closest measurements will be averaged.

Further, body composition will be assessed using bioimpedance (BIA) (101 Anniversary, Akern, Florence, Italy). BIA has been widely used in the sports field for the assessment of body composition and cellular health. BIA is not subject to errors related to prediction equations since it interprets the raw bioimpedance values [resistance (R) and reactance (c)], and it is an easy way to use and non-invasive method. BIA will be performed by a phase-sensitive single-frequency bioimpedance analyzer. Vector length (VL) and phase angle (PhA) will be calculated. This former parameter is an indicator of cellular health. Also, body fat-free mass will be estimated with BIA, while using previous validated equations. Body fat mass will be calculated by subtracting fat-free mass from total body mass. The participants will be fasting for at least 8 h, with a good night sleep, and all metallic accessories will be removed. The participants will be lying down for 5 min prior to this assessment.

Finally, two pictures of the participants will be taken (frontal and lateral), so that postural unbalances can be detected in all assessment moments. This is a simple and quick procedure to assess postural alterations.

##### Psychosocial assessments

**Fatigue and overall discomfort**

The Portuguese version of the Fatigue Scale [63] will be used to evaluate the overall level of fatigue, which comprises 8 items answered on a Likert point scale ranging from 0 (“nothing”) to 4 (“extremely”). Moreover, to specifically address the level of fatigue related to work, the Work Recovery Scale will be used, which has 11 items with dichotomic answer 0 (“no”) and 1 (“yes”). The Work Ability Index of Stanford Presenteeism Scale will be used to assess changes in the ability to work, presenteeism, and job satisfaction; it consists of an 8-item questionnaire answered in a Likert scale ranging from 0 (“no time at all”) to 4 (“the whole time”).

An adapted version of the Nordic Musculoskeletal Questionnaire will be used to assess musculoskeletal discomfort and other health symptoms on the prior week; this questionnaire includes nine regions of the body (neck, shoulders, elbows, hands/wrists, thoracic region, lumbar region, hips/thighs, knees, and ankles/feet) and quantifies pain that participants may feel in each region.

**Life/work satisfaction and quality of life**

To access participants’ perspective about their overall quality of life and satisfaction with life/work, the economic screening measure of the original World Health Organization Quality of Life Instrument-Abbreviated Version (WHOQOL-BREF) will be used. This questionnaire comprises four domains—physical, psychological, environmental, and social—with 2 items each to be answered on a 5-point Likert scale. Response adequate to each statement/item, i.e., the first item has a response from 0 (“very bad”) to 4 (“very good”), with 1 being “bad”, 2 being “not good or bad”, and 3 being “good”. For items 3 and 7, the answers range from 0 (“nothing”) to 4 (“completely”), and the remaining items ranging from 0 (“very unsatisfied”) to 4 (“very satisfied”) [64]. Moreover, participants will be asked to report how they felt in the last 2 weeks, following The World Health Organization-Five Well-being Index (WHO-5), which compresses 5 items, evaluated in a 6-point Likert scale ranging from 0 (“never”) to 5 (“all the time”) [65]. Finally, work-related well-being will be addressed using a shorter version of the original Utrecht Work Engagement Scale (UWES-9), which has 9 items evaluated in a 7-point Likert scale ranging from 0 (“never”) to 6 (“everyday”) [66].

**Eating-related habits/behaviors**

Eating-related habits/behaviors will be assessed using (a) an 11-item original questionnaire assessing the adherence to the Mediterranean diet [67], in which the participants report the weekly consumption of 9 food groups (i.e., (1) non-refined cereals, (2) fruits, (3) vegetables, (4) legumes, (5) potatoes, (6) fish, (7) meat and meat products, (8) poultry, and (9) full-fat dairy products including cheese), plus olive oil use in cooking and alcoholic beverage intake, based on the principles of the traditional Mediterranean Diet, on a scale of 0 to 5 (never, rare, frequent, very frequent, weekly, and daily consumption), and then a composite score is calculated; (b) 5 items assessing specific behaviors such as breakfast consumption, skipping meals, fast food consumption, homemade meals, and take-away meals, answered on a 4-point scale, from never to 6 or more times per week; (c) and 10 items assessing eating behavior traits: Emotional eating will be assessed with an indicator from the Intuitive Eating Scale – 2 (IES-2; “I find myself eating when I’m feeling emotional (e.g., anxious, depressed, sad), even when I’m not physically hungry”) [68]. Eating restriction will be assessed with three indicators adapted from the Dutch Eating Behavior Questionnaire (DEBQ), namely “I often refuse foods or drinks because I am concerned about my weight” (general restraint); “I watch exactly what I eat” (rigid restraint), and “When I have eaten too much, I eat less than usual the following days” (flexible restraint). Two indicators from the IES-2 will be used to measure reliance on hunger and satiety cues (“I trust my body to tell me when to eat”; “I trust my body to tell me how much to eat”), and one indicator from the same scale will measure body-food choice congruence (“I mostly eat foods that make my body perform well”) [69]. External eating will be assessed with two indicators from the Three-Factor Eating Questionnaire (TFEQ-R21), namely “Being with someone who is eating, often makes me want to also eat” and “When I smell appetizing food or see a delicious dish, I find it very difficult not to eat—even if I’ve just finished a meal”. Another indicator from the TFEQ-R21 will also be used to measure uncontrolled/disinhibited eating (“Sometimes when I start eating, I just can’t seem to stop”) [70]. Dimensions with two or more items were calculated by averaging the scores of the corresponding items. Greater values indicate greater levels on that eating trait.

#### Procedures

After complying with the inclusion criteria, participants will be contacted via email to schedule their baseline evaluation, as well as be informed in advance about the conditions of the study, and that they must attend to the evaluation morning fasting and with an empty bladder. The day before the evaluation, participants will receive an email confirming the place, date, and time of their procedure, and a reminder of all the criteria they must fulfil to be assessed.

On the scheduled day, each participant will sign the free and informed consent and will be identified through an alphanumeric code (e.g., suf01), to maintain the anonymity of their information. Initially, it will be confirmed with each participant whether they remain fasting and if they have an empty bladder (as agreed in advance), and the information will be registered in a Microsoft Excel software file®. The start of the evaluation will be by the measurement of body mass (TANITA BC-601 scale), height (Seca, Hamburg, Germany), and waist circumference (according to the World Health Organization: the midpoint between the lower border of the rib cage and the iliac crest, after a normal expiration) [71]. The height measurement will be performed with the feet slightly apart, with nearby heels, a neutral spine position, and the head according to the Frankfurt plan.

After the measurement’s procedures, we will ask participants to remove all their metal belongings to evaluate their body composition, using BIA. They must lie in the supine position for about 5 min, with their legs slightly apart and arms along the body for placement of the electrodes in the standardized points (right hand and foot) [71], thus enabling the verification of reactance and resistance values. The postural evaluation will take place then, through two photos of the evaluated standing, and in two different positions (front and side), with the feet slightly apart at the width of the hip, relaxed shoulders, and spine in a neutral position. After an explanation of how to use the ActivPAL, the participants will receive the ActivPal device, which will be placed, with the aid of a waterproof pharmacological dressing, at the right thigh's midpoint.

It will be mentioned that the participant will have to use it for 7 consecutive days (without ever taking it off) from the day of the assessment and that the device will measure their PA, SB, and sleep, so it is essential that they maintain their normal routine. It will also be ensured that the participant does not perform any aquatic activity that maintains prolonged contact with water (e.g., diving, swimming). One day before the end of the period of use of the device, an email will be sent informing them about the place and time of the return of the device. Finally, the participant will fill out an online questionnaire, and in case of any questions, one trained evaluator can immediately assist. The questionnaire was built using *Qualtrics* online software, and it is divided into several blocks that involve different themes targeted for assessment, as previously stated. The questionnaire starts by presenting the informed consent (which the participant must agree with to fill the rest of the questionnaire), followed by relevant information about the participant, including the respective ID given by the technicians, the data collected in the on-site evaluation (their height, weight, and waist circumference), and some relevant sociodemographic information, as said before. Information about participants’ health and sedentary patterns will also be measured.

### Plans to promote participant retention and complete follow-up {18b}

Assessments will be conducted by the end of the intervention period and at the end of the follow-up. To maximize retention at all assessments we will:

- Send participants an advance reminder that measurements are upcoming, using a personalized email sent 2 to 3 weeks ahead of the measurement dates, complemented by a phone call to confirm/arrange an appropriate appointment time (suited to participants schedules).
- Send a confirmation of the date, time, and location (always inside the campus of the university, in a calm and adequate location).
- Text participant in the previous day leading up to their appointment to remind them about the time, date, and location.

To enhance overall adherence to assessment periods, feedback will be provided at the end, to all participants, assuring valuable personal information regarding participants sedentary patterns, body composition, eating patterns, health and work-related outcomes, fatigue, presenteeism, job satisfaction, well-being, and quality of life, and information on how these have changed over the course of the study. The control group will only receive that feedback on the second evaluation moment, as providing feedback on the baseline can influence their actions during the control phase, which is not desired.

The value, detail, and richness of the information provided via these assessments will be highlighted since the beginning of the study, for example in the dissemination contacts/explanation of the trial, as well as in the initial psychoeducational session. Furthermore, this initial session will highlight the importance of participation in the trial (description of how important RCTs are for science will be embedded in the information provided), and thus, reducing the risk of dropout. The control group (in higher risk of dropping out in the absence of intervention) will also be provided with the opportunity of having the intervention (waiting-list group) after the 6-month assessment period.

During the trial, a website will be nurtured with interesting information (i.e., short films highlighting the evidence-based benefits of sit-stand desk usage; shortcuts highlighting the several phases of the study with the inclusion of the participants (see Intervention description {11a} for more details).

### Data management {19}

Data entry, processing, and management will be done by two researchers supervised by the PI as follows steps:

1. We will create a database with all measures obtained in data collection. An identification code will be generated for each participant to guarantee their privacy.
2. A data set will be exported and saved by Qualtrics software containing all information obtained via questionnaires.
3. We will also extract and save raw files based on ActivPAL information as well as a database will be created with PA, SB, and sleep information.
4. A New database will collate and store anonymized data securely with all domains of the project.
5. Data quality control will be accomplished by documentation checks, monitoring putative punching errors, and systematic controls.

### Confidentiality {27}

All collected data will be kept strictly confidential. The data obtained within the trial will be computerized and encrypted in a database, not containing any identifying elements of the participants. All participants will be given a unique ID number, nor by name (apart from the consent inform). This database will be maintained by those responsible for the investigation, on a secure server of CIDEFES-UL, for 10 years for research purposes only.

### Plans for collection, laboratory evaluation, and storage of biological specimens for genetic or molecular analysis in this trial/future use {33}

Not applicable.

## Statistical methods

### Statistical methods for primary and secondary outcomes {20a}

Statistical analysis will be performed using PASW Statistics for Windows version 28.0 (SPSS Inc., an IBM Company, Chicago IL, USA). Data will be analyzed for those having completed pre–post test data and using an intention-to-treat analysis. Baseline differences between the intervention and control group will be examined using independent samples *t*-tests. If exploratory analysis reveals a non-normally distribution for some variables, it will be alternatively used with the non-parametric tests. Repeated measures ANOVA, with time (baseline-M0, M1, and M2) as within-subjects factor and intervention condition as between-subjects factor, will be conducted to evaluate the effects of the intervention on primary and on secondary outcomes. Statistical significance will be set at *p* < 0.05.

### Interim analyses {21b}

The intervention was considered low risk, and therefore, interim analyses and formal stopping rules were not deemed necessary by the research team. However, the study protocol will include monitoring for adverse events via an online form that will be periodically reviewed to ensure participant safety.

### Methods for additional analyses (e.g., subgroup analyses) {20b}

Not applicable. We will probably not have enough sample size to allow subgroup analyses.

### Methods in analysis to handle protocol non-adherence and any statistical methods to handle missing data {20c}

Other than the intention-to-treat procedures, we will opt not to use any statistical artifact to handle missing data. We will, however, include more participants than needed (40 vs 34), anticipating some dropout or missing data during the trial.

### Plans to give access to the full protocol, participant-level data, and statistical code {31c}

Public access to the full protocol is granted by the registration in a public online platform https://doi.org/10.17605/OSF.IO/JHGPW and also by the SUFHA website http://sufha.ulusofona.pt/.

## Oversight and monitoring

### Composition of the coordinating center and trial steering committee {5d}

A Steering Committee will be formed at the beginning of this study for monitoring the trial. This committee will be led by the PI, in a small group to run smoothly in monitoring the activities under trial. The Steering Committee will meet every week with the research team to analyze the study progressions and to propose counter measures when deviations occur from the established trial plan.

### Composition of the data monitoring committee, its role and reporting structure {21a}

A data monitoring committee will not be needed for the SUFHA trial, given that both interventions are non-invasive with minimal risk of harm. Regardless, the research team will meet once a week to discuss participants and intervention progress, emerging challenges and required adjustments to the protocol.

### Adverse event reporting and harms {22}

Although the known risks of using sit-stand desks are low, SUFHA has the following procedures to report such events.

An adverse event (AE) is any negative event reported by a participant or observed by the investigator during the trial, which may not be related to the intervention. All AEs will be recorded in a form by the research team with information about severity, frequency, date of onset, duration, and action taken, as well as the relationship between the AE and the intervention and the participant outcome. Participants will be instructed to communicate to the research team any discomfort or unexpected adverse reaction to using the sit-stand desk.

### Frequency and plans for auditing trial conduct {23}

The research team will meet regularly once a week to discuss participant and intervention progress, emerging challenges, and required adjustments to the protocol. These meetings will also serve to monitor data on outcomes and adverse events, and to oversee participants’ safety. A data monitoring committee will not be needed for the SUFHA trial, given that the interventions are non-invasive with minimal risk of harm.

### Plans for communicating important protocol amendments to relevant parties (e.g., trial participants, ethical committees) {25}

If any changes to the protocol are needed, our plan is to first notify the sponsor and funder. Then, the principal investigator (PI) will notify the ethical commission and send a copy of the revised protocol to be added to the research center CIDEFES project management database. Any deviations from the protocol will be fully documented under a report sent to the CIDEFES. This way, we can maintain transparency and accountability throughout the study.

Finally, we will update the protocol in the clinical trial registry to ensure that all stakeholders have access to accurate and up-to-date information about the study.

### Dissemination plans {31a}

The rationale for this project entails novelty and generates new knowledge enough to publish at least 3 scientific papers in international peer-review Journals. (1) protocol publication; (2) main results from the intervention considering the several outcomes; (3) main results from the follow-up phase.

Furthermore, these findings will be initially presented at international and national conferences. Additionally, to disseminate the project on a larger scale, there is a plan to develop four short cut videos depicting the several phases of the project. These videos will be created by the HEI-Lab researchers, along with bachelor and master students from their faculty, and shared with the Lusófona University. The goal is to highlight the chronology of the project, and to stimulate the uptake of this type of intervention by disseminating participants’ experiences as they were shaped by the project: The first clip will show the participants in the baseline assessments. The second video will highlight the initial session. The third clip, released on the fourth month of the project, will focus on the intervention and the new reality of the participants. The fourth clip, released towards the end of the sixth month of intervention, will focus on the main changes the participants feel from a longer use of the sit-stand desks and other aspects not mentioned in the prior video. Finally, a fifth clip will be released in the follow-up period and will feature the study’s main findings and testimonies from the participants, including adaptations made.

## Discussion

Albeit the strong evidence on the health benefits of active lifestyles, an alarming proportion of adults continues with sedentary lifestyles, with an increasing trend in the prevalence of SB [72]. SB is something particularly worrying, considering that worldwide, adults spend more than 60% of their waking time in a sitting position, with most of that time taking place during work [73, 74]. To counteract this trend in office workers, it is important to apply strategies so that workers can reduce the time spent sitting and increase the time in a standing posture, using short and active breaks during their working hours [75]. One potential solution is the introduction of sit-stand desks in the workplace. Although scientific evidence is limited on this topic, sit-stand desks seem to reduce SB among office workers [76, 77] and consequently they may improve worker’s health, without compromising productivity and concentration [76, 78].

This paper describes the protocol of the SUFHA, a study designed to assess the effectiveness of a 6-month sit-stand desk workplace intervention offered to university staff, to reduce prolonged sitting and increase standing and interruptions in sitting time.

Besides the introduction of a contextual change in the workplace (i.e., sit-stand desk), this intervention will use an initial psychoeducational session to all participants, to inform about the harms of spending too much time sitting and the independent health benefits of interrupting this behavior more often. Furthermore, a motivational system of prompts will also be offered, nudging participants to stand more often, for 6 months. After the initial 6 months of the intervention, the control group will receive the sit-stand desk, and the intervention group will be without the stimulus, to observe if the new habits gained during the intervention period somehow remain after 3 months of follow-up (i.e., without the sit-stand desks). A major strength of the SUFHA is that it builds on previous interventions using sit-stand desks around the globe to target office workers in a university setting. Previous interventions have been combining sit-stand desk in addition to other strategies (i.e., multicomponent interventions) such as the usage of pedometer and/or competition among participants, or other strategies [79, 80], which makes it difficult to isolate the impact of simply using these sit-stand desks; SUFHA intends to fill this gap and extending it by providing data on maintenance of the new habits. Furthermore, feedback on the barriers and facilitators of the use of the sit-stand desks will also be collected via focus groups exploring the experiences of the participants during the trial.

The findings from this cluster RCT will provide insight for future research studies, as well as real-world interventions, and offer evidence for policy guidelines around workplace health and well-being.

The current RCT intends to inform employers and nudge them to invest in their occupational health conditions by providing evidence on the impact of using sit-stand desks. Furthermore, a refined, low-contact intervention informed by participants’ experiences will be available to be disseminated and implemented in diverse occupational settings, including the health sector, raising awareness for the positive impact of the use of sit-stand desks in multiple work settings. We hope that project findings and materials can help to nudge public and private companies from all sectors (e.g., education, health, industry, services) to adapt their work settings to use sit-stand desks, thus translating research into the “real world.” We anticipate important health benefits from this intervention, including improvements in eating patterns, as well as gains in work engagement that may convince companies and governments to invest in these new approaches, which will nurture the sustainable implementation of preventive interventions tailored to the work context, improving employee’s agency, movement behaviors, and well-being to ultimately halt the rise of NCDs and associated healthcare costs.

## Trial status

The current protocol version is dated 11/2/2023. The recruitment began on the 1st of January 2023 and ended by the end of February 2023. On the 1st of March, the intervention kicked off and is currently ongoing. Two more moments of assessment will still occur. Due to some delay in the revision of the final draft of the manuscript, we were not able to submit the manuscript prior to the last participant being recruited.

## Data Availability

Anonymized trial data will be available for non-commercial research purposes only upon request to the PI.

## Abbreviations

BDNF: Brain-derived neurotrophic factor
BMI: Body mass index
CONSORT: The Consolidation Standards of Reporting Trials
CVD: Cardiovascular diseases
DEBQ: Dutch Eating Behavior Questionnaire
LIPA: Light-intensity physical activity
PA: Physical activity
PhA: Phase angle
PI: Principle investigator
RCT: Randomized controlled trial
SB: Sedentary behavior
TFEQ-R21: Three-Factor Eating Questionnaire
UK: United Kingdom
USA: United States of America
VL: Vector length
WHO: World Health Organization
WHOQOL-BREF: World Health Organization Quality of Life Instrument-Abbreviated Version
